# Health Care Utilization in the 6 Months Following SARS-CoV-2 Infection

**DOI:** 10.1001/jamanetworkopen.2022.25657

**Published:** 2022-08-12

**Authors:** Sara Y. Tartof, Deborah E. Malden, In-Lu Amy Liu, Lina S. Sy, Bruno J. Lewin, Joshua T. B. Williams, Simon J. Hambidge, Jonathan D. Alpern, Matthew F. Daley, Jennifer C. Nelson, David McClure, Ousseny Zerbo, Michelle L. Henninger, Candace Fuller, Eric Weintraub, Sharon Saydah, Lei Qian

**Affiliations:** 1 Department of Research & Evaluation, Kaiser Permanente Southern California, Pasadena; 2 Department of Health Systems Science, Kaiser Permanente Bernard J. Tyson School of Medicine, Pasadena, California; 3 Centers for Disease Control and Prevention, Epidemic Intelligence Service, Atlanta, Georgia; 4 Denver Health, Office of Research Ambulatory Care Services, Denver, Colorado; 5 HealthPartners Institute, Bloomington; 6 Kaiser Permanente Colorado, Institute for Health Research, Denver, Colorado; 7 Kaiser Permanente Washington Health Research Institute, Seattle; 8 Marshfield Clinic Research Institute, Marshfield, Wisconsin; 9 Department of Research, Kaiser Permanente Northern California, Oakland; 10 Kaiser Permanente Northwest, Center for Health Research, Portland, Oregon; 11 Harvard Pilgrim Health Care Institute, Boston, Massachusetts; 12 Centers for Disease Control and Prevention, Immunization Safety Office, Atlanta, Georgia; 13 Division of Viral Diseases, Respiratory Viruses Branch, Centers for Disease Control and Prevention, Atlanta, Georgia

## Abstract

**Importance:**

After SARS-CoV-2 infection, many patients present with persistent symptoms for at least 6 months, collectively termed *post-COVID conditions* (PCC). However, the impact of PCC on health care utilization has not been well described.

**Objectives:**

To estimate COVID-19–associated excess health care utilization following acute SARS-CoV-2 infection and describe utilization for select PCCs among patients who had positive SARS-CoV-2 test results (including reverse transcription–polymerase chain reaction and antigen tests) compared with control patients whose results were negative.

**Design, Setting, and Participants:**

This matched retrospective cohort study included patients of all ages from 8 large integrated health care systems across the United States who completed a SARS-CoV-2 diagnostic test during March 1 to November 1, 2020. Patients were matched on age, sex, race and ethnicity, site, and date of SARS-CoV-2 test and were followed-up for 6 months. Data were analyzed from March 18, 2021, to June 8, 2022.

**Exposure:**

SARS-CoV-2 infection.

**Main Outcomes and Measures:**

Ratios of rate ratios (RRRs) for COVID-19-associated health care utilization were calculated with a difference-in-difference analysis using Poisson regression models. RRRs were estimated overall, by health care setting, by select population characteristics, and by 44 PCCs. COVID-19–associated excess health care utilization was estimated by health care setting.

**Results:**

The final matched cohort included 127 859 patients with test results positive for SARS-CoV-2 and 127 859 patients with test results negative for SARS-CoV-2. The mean (SD) age of the study population was 41.2 (18.6) years, 68 696 patients in each group (53.7%) were female, and each group included 66 211 Hispanic patients (51.8%), 9122 non-Hispanic Asian patients (7.1%), 7983 non-Hispanic Black patients (6.2%), and 34 326 non-Hispanic White patients (26.9%). Overall, SARS-CoV-2 infection was associated with a 4% increase in health care utilization over 6 months (RRR, 1.04 [95% CI, 1.03-1.05]), predominantly for virtual encounters (RRR, 1.14 [95% CI, 1.12-1.16]), followed by emergency department visits (RRR, 1.08 [95% CI, 1.04-1.12]). COVID-19–associated utilization for 18 PCCs remained elevated 6 months from the acute stage of infection, with the largest increase in COVID-19–associated utilization observed for infectious disease sequelae (RRR, 86.00 [95% CI, 5.07-1458.33]), COVID-19 (RRR, 19.47 [95% CI, 10.47-36.22]), alopecia (RRR, 2.52 [95% CI, 2.17-2.92]), bronchitis (RRR, 1.85 [95% CI, 1.62-2.12]), pulmonary embolism or deep vein thrombosis (RRR, 1.74 [95% CI, 1.36-2.23]), and dyspnea (RRR, 1.73 [95% CI, 1.61-1.86]). In total, COVID-19-associated excess health care utilization amounted to an estimated 27 217 additional medical encounters over 6 months (212.9 [95% CI, 146.5-278.4] visits per 1000 patients).

**Conclusions and Relevance:**

This cohort study documented an excess health care burden of PCC in the 6 months after the acute stage of infection. As health care systems evolve during a highly dynamic and ongoing global pandemic, these data provide valuable evidence to inform long-term strategic resource allocation for patients previously infected with SARS-CoV-2.

## Key Points

**Question:**

Is SARS-CoV-2 associated with health care utilization 6 months after the acute stage of infection?

**Findings:**

In this cohort study of 127 859 patients with positive SARS-CoV-2 test results matched to 127 859 patients with negative SARS-CoV-2 test results, health care utilization was elevated in patients with positive SARS-CoV-2 results 6 months after the acute infection. Other than COVID-19 and infectious disease sequelae, the most notable post–COVID-19 conditions associated with elevated health care utilization over 6 months included alopecia (hair loss), bronchitis, pulmonary embolism or deep vein thrombosis, and dyspnea.

**Meaning:**

These findings suggest that health care systems should consider long-term strategic resource allocation in response to the expected elevated health care utilization experienced by patients with SARS-CoV-2 infection for at least 6 months following the acute stage of infection.

## Teaser

This cohort study examines excess health care utilization associated over the course of 6 months after SARS-CoV-2 infection compared with individuals with negative SARS-CoV-2 test results.

## Introduction

COVID-19 has contributed to more than 5.9 million deaths globally, as of February 2022. The economic cost of premature deaths alone is estimated to exceed $4 trillion in the United States through 2021.^1^ In health care settings, management of the acute phase of infection has been prioritized for resource allocation. Yet, a proportion of patients develop persistent and debilitating symptoms across multiple organ systems lasting longer than 2 to 4 weeks from the onset of COVID-19 symptoms.^2^ The long-term effects of SARS-CoV-2 infection are often referred to as *post-COVID conditions* (PCC) or *post-acute sequelae of COVID-19* and can occur despite relatively mild illness at onset.^3,4^

While there has been a recent increase in published research on PCC, investigating the true clinical manifestations has been challenging owing to the complex and varied nature of symptoms and limitations of study designs to date.^2,5,6,7^ Some of the most robust evidence characterizing PCC has been generated by a number of population studies^8,9,10,11,12,13^; however, most have lacked appropriate control populations.^7,11,12,13,14,15^ Additionally, while some studies have attempted to control for changes in health care utilization during the pandemic^16^ or systematic differences in population characteristics between individuals who contract COVID-19-those who do not,^17^ few have considered these biases simultaneously. Moreover, study populations are often limited to adult patients or patients requiring hospitalization,^13,14,17,18^ compromising the generalizability of the findings.

This study combines data from 8 geographically diverse integrated health care organizations across the United States to evaluate PCC in a large, demographically and clinically diverse population of all ages across all care settings (ie, outpatient, inpatient, emergency department [ED], and virtual settings). We leveraged this nationally representative cohort to estimate overall health care utilization after acute COVID-19 and describe excess utilization for select PCC diagnoses among patients with test results positive for SARS-CoV-2 compared with control patients with negative results.^19^

## Methods

This cohort study was approved by institutional review boards at each participating site with waivers of the Health Insurance Portability and Accountability Act and informed consent, as the data-only research activities were determined to pose minimal risk. This study conforms to the Strengthening the Reporting of Observational Studies in Epidemiology (STROBE) reporting guideline.

### Study Setting

This study was conducted within the Vaccine Safety Datalink (VSD), a research collaboration led by the Centers for Disease Control and Prevention (CDC) that combines electronic medical record (EMR) databases to conduct large epidemiological studies.^19,20^ Eight VSD sites contributed data: Kaiser Permanente Southern California (lead site), Denver Health, HealthPartners Institute, Kaiser Permanente Colorado, Kaiser Permanente Northern California, Kaiser Permanente Northwest, Kaiser Permanente Washington, and Marshfield Clinic Research Institute.

### Study Design

This matched retrospective cohort study included patients with a SARS-CoV-2 diagnostic test for any indication, regardless of symptoms. We used a difference-in-difference design to compare the change in utilization for select outcomes in the population with test results positive for SARS-CoV-2 (exposure group) vs the change in outcomes in the population with test results negative for SARS-CoV-2 (comparison group), before and after the global COVID-19 pandemic was declared. By comparing health care utilization within the same individuals during 2 time periods (2019 and 2020), we controlled for individual-level variables, such as demographics, health status, and health care–seeking behavior. By adding the negative SARS-CoV-2 test results comparison group, adjustments for secular confounding, such as changes to health care delivery due to the COVID-19 pandemic, were incorporated as well.

### Study Population

The study cohort was identified from the population of patients of all ages receiving care from the 8 study sites from March 15, 2019, to May 15, 2021. The positive SARS-CoV-2 test results exposure group was defined as patients with a positive result in laboratory testing for SARS-CoV-2 from March 1, 2020, to November 1, 2020 (including reverse transcription–polymerase chain reaction and antigen tests). Each patient with positive SARS-CoV-2 results was matched 1:1 with a randomly selected patient with negative test results, defined as having a negative result on a diagnostic test from March 1, 2020, to November 1, 2020. Matching was conducted based on VSD site, age (±5 years), sex (male or female), self-reported race and ethnicity (categorized as Hispanic, non-Hispanic Asian, non-Hispanic Black, non-Hispanic White, and multiple, other [including individuals who identified as American Indian or multiple or other race and ethnicity], or unknown), and date of test (±10 days). Race and ethnicity were included because they are associated with both the exposure (vaccination) and the outcome (health care utilization).

The index date was defined as the 14th day following the date of the positive or negative SARS-CoV-2 laboratory test result to allow for a 2-week washout period for acute illness. A 6-month postindex period and a 6-month preindex period exactly 1 year prior to the postindex period was defined for each patient. For example, if a patient had test results positive for SARS-CoV-2 on May 1, 2020, then May 15, 2020, was the assigned index date; May 15 to November 15, 2020, was the postindex period; and May 15 to November 15, 2019, was the preindex period. This logic was similarly applied to the patients with negative SARS-CoV-2 test results, anchored on their index date. Preindex periods included March 15, 2019, to May 15, 2020, and postindex periods included March 15, 2020, to May 15, 2021.

Continuous health care organization membership (allowing for a 31-day administrative gap for membership renewal) was required from 12 months prior to the index date to 6 months after the index date to ensure comprehensive assessment of utilization and comorbidity data. The capture of care was considered highly complete for all integrated health care organizations contributing data. Medical care delivered outside of these systems was captured in the system through claims that were required for reimbursement.

### Outcomes

Utilization rates associated with health care encounters during the 6-month preindex period and 6-month postindex period were calculated by dividing the number of encounters by the number of patients per stratum. Utilization rates associated with 44 PCC diagnoses selected in consultation with the CDC (eTable 1 in the Supplement) were also calculated, overall and by setting (ie, outpatient, inpatient, ED, and virtual), time interval (ie, first 3 months and 4-6 months following index date), and age group (ie, <18 years and ≥18 years). Inpatient outcomes with admission dates that occurred at or after the index date were included.

### Covariates

Data were collected on demographic characteristics, including age, sex, and race and ethnicity. Clinical comorbidities according to the CDC’s definition of COVID-19 high-risk categories were collected from the EMR in the year prior to index date^20,21^ (including 2475 EMR codes; list available on request). Additionally, we collected information on COVID-19 vaccination during follow-up, defined as any documentation of receipt of an mRNA COVID-19 vaccine (Pfizer-BioNTech or Moderna) or viral vector (Johnson & Johnson) vaccine in the 6 months after the index date.

### Statistical Analysis

Baseline clinical and demographic characteristics were described and compared between groups with positive or negative SARS-CoV-2 test results. Continuous variables were summarized by mean, SD, median, IQR, and range and compared using Wilcoxon signed-rank tests; categorical variables were summarized by frequency distributions and compared using χ^2^ tests or Fisher exact test.

For overall utilization and utilization associated with each PCC outcome, we estimated the ratio of the encounter rate during the postindex period compared with the preindex period for the population with positive SARS-CoV-2 test results and matched population with negative results. The difference-in-difference parameter was calculated as the adjusted increase in encounter rate from postindex vs preindex periods (ie, ratios of rate ratios [RRRs] and 95% CIs) associated with SARS-CoV-2 positivity by fitting robust Poisson regression models with an interaction term of time period and positive or negative test result group. Estimates for overall utilization were generated by setting (outpatient, inpatient, ED, and virtual), age group, sex, race and ethnicity, and select comorbidities. Estimates of utilization for each PCC were generated for all patients and separately for patients aged 0 to 17 years and by time interval (first 3 months and 4-6 months after index date). Where certain PCC outcomes displayed unusually strong associations among children, potential cases underwent EMR review by a clinical expert (B.J.L.) to verify the diagnosis. Sensitivity analyses were conducted by excluding follow-up time at the date of receipt of COVID-19 vaccine, as well as the corresponding preindex period of the vaccinated patient and preindex and postindex periods of vaccination for matched patient pairs.

The numbers of excess visits associated with COVID-19 were calculated as the difference of health care utilization among patients with test results positive for SARS-CoV-2 (n) and the utilization among the same group of patients if they were not infected (*n* / *RRR*), where *n* is number of visits during 6 months of follow up after infection among patients with positive SARS-CoV-2 test results. The volumes of visits were estimated overall, by outcome, and by health care setting.

*P* values were 2-sided, and statistical significance was set at *P* = .05. Statistical analyses were conducted using SAS software version 9.4 (SAS Institute). Data were analyzed from March 18, 2021, to June 8, 2022.

## Results

The final matched cohort consisted of 127 859 patients with positive SARS-CoV-2 test results and 127 859 patients with negative SARS-CoV-2 test results, with a mean (SD) age of 41.2 (18.6) years (Table 1). Most patients (251 592 patients [98.4%]) were tested via PCR (vs antigen tests: 4126 patients [1.6%]). More than half of all participants (137 392 of 255 718 patients [53.7%]) were female. Each group included 66 211 Hispanic patients (51.8%), 9122 non-Hispanic Asian patients (7.1%), 7983 non-Hispanic Black patients (6.2%), and 34 326 non-Hispanic White patients (26.9%). Common comorbidities included hypertension (46 606 patients [18.2%]), overweight or obesity (46 132 patients [18.0%]), and diabetes (31 477 patients [12.3%]). Comorbidities were more common among patients with negative SARS-CoV-2 test results than those with positive SARS-CoV-2 test results, except for diabetes, overweight or obesity, and neurologic conditions (Table 1). A total of 156 patients with positive SARS-CoV-2 results tested over the study period were excluded from the analyses owing to a lack of matched controls.

**Table 1.** Characteristics of SARS-CoV-2 Positive and Matched SARS-CoV-2 Negative Populations

Characteristic	Patients by SARS-CoV-2 test result, No. (%)		*P* value
	Negative (n = 127 859)^a^	Positive (n = 127 859)	
Age at index date, y			
Mean (SD)	41.2 (18.6)	41.2 (18.6)	NA
Median (IQR) [range]	40.9 (27.3-54.8) [1.0-105.8]	40.9 (27.3-54.8) [1.0-108.9]	NA
0-2	1109 (0.9)	1110 (0.9)	>.99
3-17	12 433 (9.7)	12 459 (9.7)	
18-49	71 305 (55.8)	71 274 (55.7)	
50-64	29 697 (23.2)	29 726 (23.3)	
65-84	12 239 (9.6)	12 205 (9.6)	
≥85	1076 (0.8)	1085 (0.9)	
Sex			
Female	68 696 (53.7)	68 696 (53.7)	>.99
Male	59 163 (46.3)	59 163 (46.3)	
Race and ethnicity			
Hispanic	66 211 (51.8)	66 211 (51.8)	>.99
Non-Hispanic			
Asian	9122 (7.1)	9122 (7.1)	
Black	7983 (6.2)	7983 (6.2)	
White	34 326 (26.9)	34 326 (26.9)	
Multiple, other, or unknown^b^	10 217 (8.0)	10 217 (8.0)	
Comorbid conditions			
COPD	2264 (1.8)	1652 (1.3)	<.001
Cancer	3896 (3.1)	2362 (1.9)	<.001
Chronic kidney disease	4492 (3.5)	3830 (3.0)	<.001
Heart conditions	5708 (4.5)	4600 (3.6)	<.001
Immunocompromised	2312 (1.8)	1713 (1.3)	<.001
Sickle cell disease	38 (0.03)	12 (0.01)	<.001
Diabetes	14 633 (11.4)	16 844 (13.2)	<.001
Overweight or obesity	22 616 (17.7)	23 516 (18.4)	<.001
Down syndrome	65 (0.1)	52 (0.04)	.23
Asthma	1326 (1.0)	1070 (0.8)	<.001
CVD	2099 (1.6)	1721 (1.4)	<.001
Cystic fibrosis	20 (0.02)	7 (0.01)	.01
Hypertension	23 683 (18.5)	22 923 (17.9)	<.001
Liver disease	4501 (3.5)	3836 (3.0)	<.001
Pulmonary fibrosis	516 (0.4)	404 (0.3)	<.001
Thalassemia	105 (0.1)	81 (0.1)	.08
Neurologic conditions	824 (0.6)	1159 (0.9)	<.001
Test month in 2020			
March	2527 (2.0)	2532 (2.0)	.82
April	5785 (4.5)	5795 (4.5)	
May	5798 (4.5)	5798 (4.5)	
June	18 080 (14.1)	18 061 (14.1)	
July	40 733 (31.9)	40 699 (31.8)	
August	18 448 (14.4)	18 479 (14.5)	
September	13 896 (10.9)	13 898 (10.9)	
October	22 204 (17.4)	22 149 (17.3)	
November	388 (0.3)	448 (0.4)	
Site			
A	40 405 (31.6)	40 405 (31.6)	>.99
B	4203 (3.3)	4203 (3.3)	
C	4361 (3.4)	4361 (3.4)	
D	5668 (4.4)	5668 (4.4)	
E	4148 (3.2)	4148 (3.2)	
F	2951 (2.3)	2951 (2.3)	
G	64 119 (50.2)	64 119 (50.2)	
H	2004 (1.6)	2004 (1.6)	
COVID-19 vaccine doses during follow-up, No.^c^			
0	98 225 (76.8)	104 594 (81.8)	<.001
1	11 583 (9.1)	9486 (7.4)	
2	17 970 (14.1)	13 692 (10.7)	
3	65 (0.1)	77 (0.1)	
≥4	16 (<0.1)	10 (<0.1)	

Abbreviations: COPD, chronic obstructive pulmonary disease; CVD, cerebrovascular disease; NA, not applicable.

^a^ Matching was 1:1 based on VSD site, age (±5 years), sex (male or female), race and ethnicity, and date of test (±10 days).

^b^ Includes individuals who identified as American Indian or other races and ethnicities.

^c^ COVID-19 vaccine dose count was defined as number of documented mRNA COVID-19 vaccines (Pfizer-BioNTech or Moderna) or virus vector (Johnson & Johnson) vaccines received in the 6 months after the index date.

### Overall Health Care Utilization

**Figure 1.**
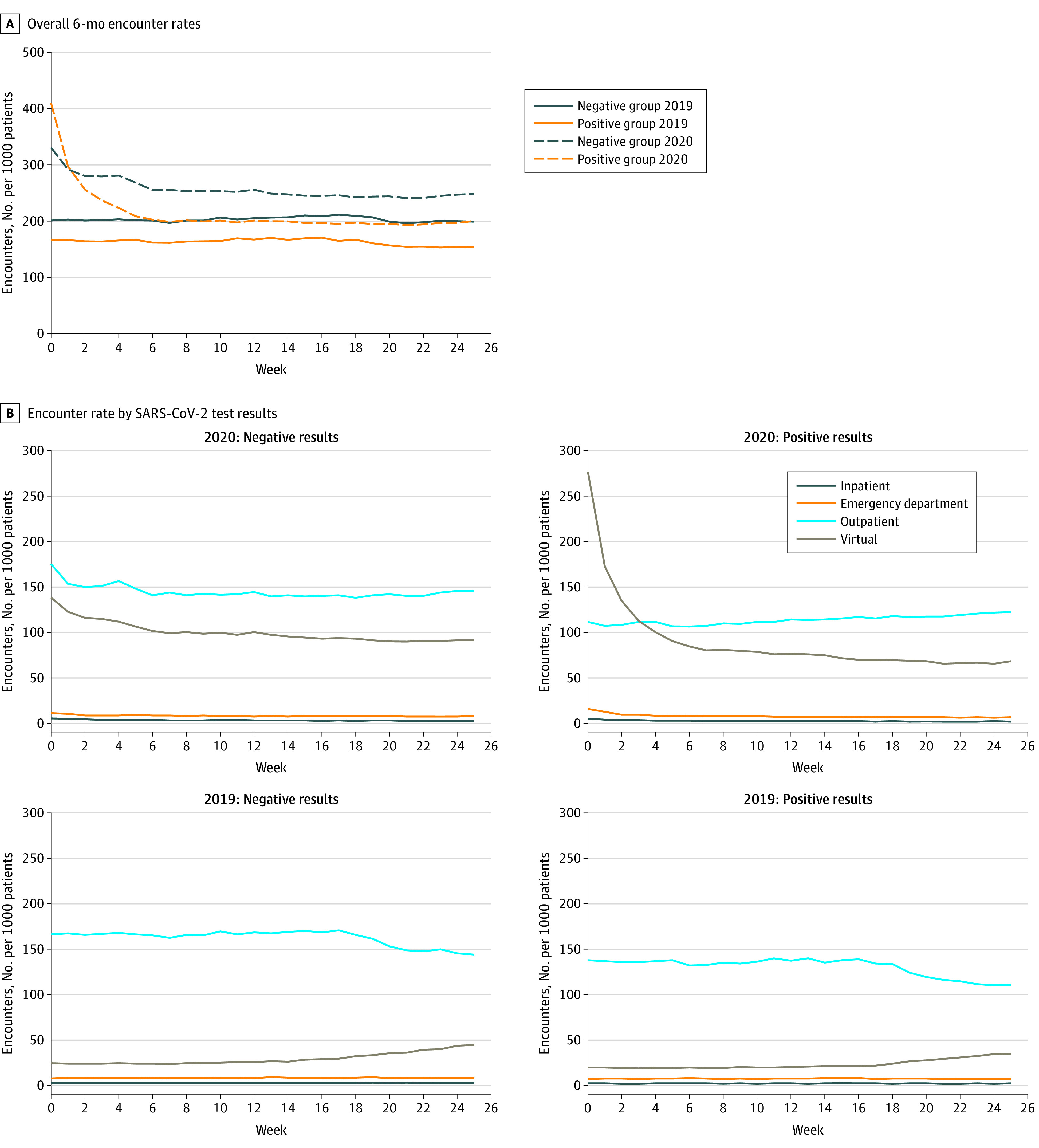
Weekly Health Care Encounter Rate 6 Months Before and After SARS-CoV-2 Testing

During the preindex period in 2019, the population with negative SARS-CoV-2 test results had consistently higher health care utilization than the population with positive SARS-CoV-2 test results (Figure 1A). In the postindex period in 2020, the group with positive SARS-CoV-2 test results had higher overall health care utilization rates in the first week following the acute stage of infection (approximately 3 weeks after date of SARS-CoV-2 test), followed by higher health care utilization rates from weeks 2 to 25 in the group with negative SARS-CoV-2 test results. The highest utilization rates in 2020 for the population with negative SARS-CoV-2 test results occurred in the outpatient setting, followed by the virtual setting; however, health care utilization in both settings declined during the first 7 weeks after the index date (Figure 1B). The secular patterns by setting were similar between groups in the 2019 preindex period (Figure 1B). During the 2020 period among patients with positive SARS-CoV-2 test results, virtual care was highly used in the immediate weeks after the index date but declined rapidly after 6 weeks. Outpatient care became more common than virtual care after approximately 3 weeks among patients with positive SARS-CoV-2 test results.

**Figure 2.**
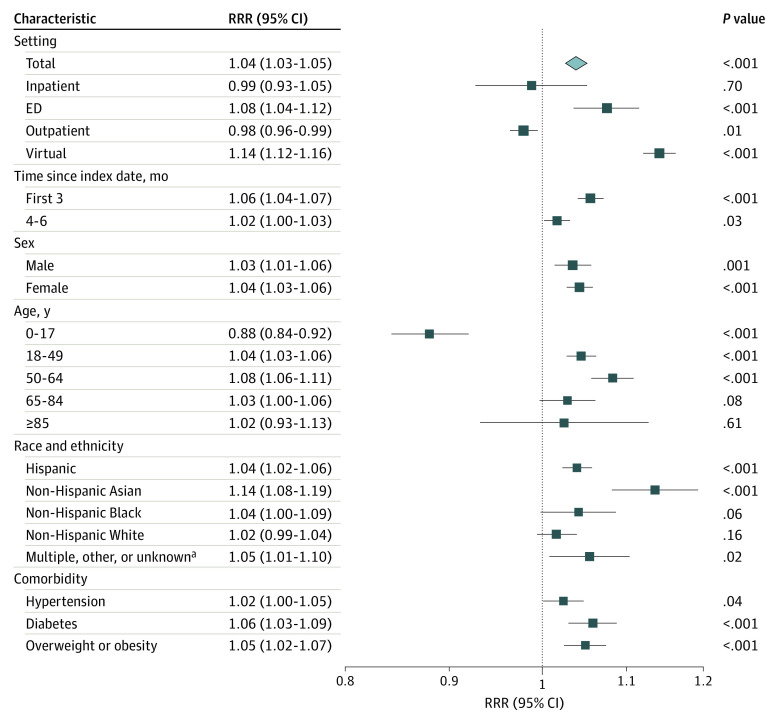
Health Care Utilization Associated With Positive SARS-CoV-2 Test Results vs Negative SARS-CoV-2 Test Results The difference-in-difference parameter was calculated as the adjusted increase in encounter rate from posttest vs pretest periods associated with SARS-CoV-2 positivity by fitting Poisson regression models with robust error variance. RRR indicates relative rate ratio. ^a^Includes individuals who identified as American Indian or other or multiple races and ethnicities.

Difference-in-difference analyses comparing populations by SARS-CoV-2 test results demonstrated an overall increase of 4% in health care utilization over 6 months among patients with positive test results for SARS-CoV-2 (RRR, 1.04 [95% CI, 1.03-1.05]) (Figure 2). By setting, COVID-19–associated increases in health care utilization were highest for virtual encounters (RRR, 1.14 [95% CI, 1.12-1.16]), followed by ED encounters (RRR, 1.08 [95% CI, 1.04-1.12]); outpatient encounters also declined (RRR, 0.98 [95% CI, 0.96-0.99]). Increased overall utilization associated with COVID-19 was highest in the first 3 months (RRR, 1.06 [95% CI, 1.04-1.07]) but remained elevated at 4 to 6 months (RRR, 1.02 [95% CI, 1.00-1.03]). Compared with adults aged 18 years and older, COVID-19 among children was associated with lower overall health care utilization over 6 months (RRR, 0.88 [95% CI, 0.84-0.92]). Compared with all other race and ethnicities, Asian patients with positive SARS-CoV-2 test results had the highest relative increase in utilization (RRR, 1.14 [95% CI, 1.08-1.19]).

### Conditions of Interest

**Figure 3.**
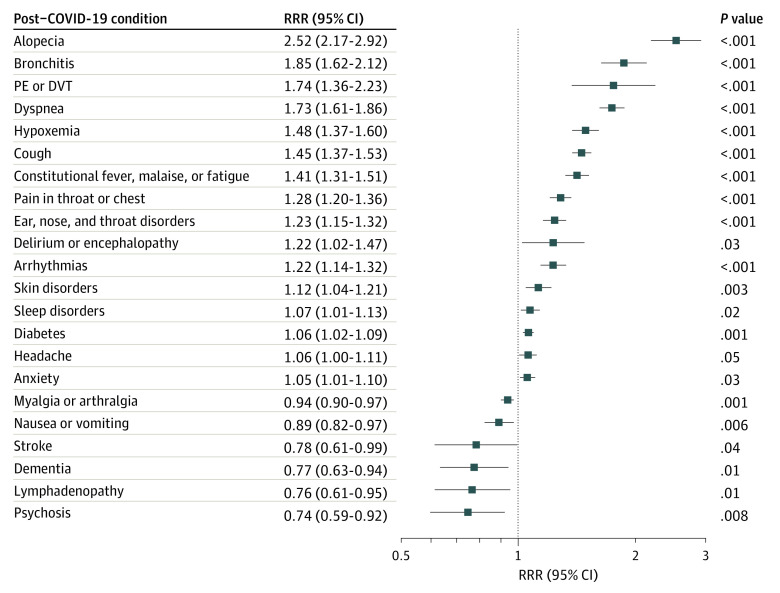
Health Care Utilization Associated With COVID-19 for Select Post–COVID-19 Conditions Compared With Patients with Negative SARS-CoV-2 Test Results Outcomes presented are limited to those conditions that were statistically significant overall over 6 months (24 of 44 conditions evaluated), excluding infectious disease sequelae (relative rate ratio [RRR], 86.00; 95% CI, 5.07-1458.33) and COVID-19 (RRR, 19.47; 95% CI, 10.47-36.22), owing to scale. DVT indicates deep vein thrombosis; PE, pulmonary embolism.

Of 44 established PCC diagnosis categories, 24 were significantly associated with COVID-19 at 6 months after the acute stage of disease (Figure 3; eTable 2 in the Supplement). Notable PCCs identified with increased COVID-19–associated health care utilization after 6 months included COVID-19 (RRR, 19.47 [95% CI, 10.47-36.22]), alopecia (RRR, 2.52 [95% CI, 2.17-2.92]), bronchitis (RRR, 1.85 [95% CI, 1.62-2.12]), pulmonary embolism (PE) or deep vein thrombosis (DVT) (RRR, 1.74 [95% CI, 1.36-2.23]), dyspnea (RRR, 1.73 [95% CI, 1.61-1.86]), hypoxemia (RRR, 1.48 [95% CI, 1.37-1.60]), and infectious disease sequelae, although CIs were wide around this estimate (RRR, 86.00 [95% CI, 5.07-1458.33]). Of all 44 PCCs, 20 were significantly associated with COVID-19 after 3 months, and 16 were significantly associated with COVID-19 at 4 to 6 months after acute illness (eFigure 1 in the Supplement). Of note, some PCCs were associated with higher utilization during early periods (0-3 months) compared with later periods (4-6 months) (eg, bronchitis; cough; constitutional fever, malaise, or fatigue; hypoxemia, pain in throat or chest; and dyspnea), some were persistently elevated throughout follow-up (ie, alopecia, PE or DVT, ear, nose and throat [ENT] disorders, and arrythmias), and others were elevated at later time periods (4-6 months) compared with earlier time periods (0-3 months) after acute illness (ie, skin disorders, sleep disorders, and myoneural disorders). Censoring at COVID-19 vaccination during follow-up did not alter the associations with PCC outcomes (eFigure 2 in the Supplement).

Among patients younger than 18 years, COVID-19 was associated with significantly elevated health care utilization for COVID-19 (RRR, 24.07 [95% CI, 1.49-389.01), PE or DVT (RRR, 24.00 [95% CI, 1.91-301.67]), arrhythmias (RRR, 1.78 [95% CI, 1.06-2.98]), dyspnea (RRR, 1.43 [95% CI, 1.09-1.88]), and ENT disorders (RRR, 1.25 [95% CI, 1.02-1.55]). Given the high utilization rates for PE or DVT, a sample of medical records for PE or DVT diagnoses were reviewed. The diagnoses of PE or DVT were confirmed for all occurrences; however, these children appeared to have underlying conditions that may have predisposed them to PE or DVT. Four of 44 PCCs demonstrated significantly lower rates of health care utilization in children with positive SARS-CoV-2 results, including lymphadenopathy (RRR, 0.53 [95% CI, 0.30-0.95]), myalgia or arthralgia (RRR, 0.74 [95% CI, 0.60-0.91]), dementia (RRR, 0.19 [95% CI, 0.05-0.68]), and psychosis (RRR, 0.19 [95% CI, 0.06-0.59]); however, CIs were wide (eFigure 3 in the Supplement).

### Excess Health Care Utilization

We estimated an additional 27 217 health care visits were attributed to COVID-19 over 6 months (212.9 [95% CI, 146.5-278.4] visits per 1000 patients). The estimated COVID-19–associated excess visits for notable PCCs included: 53 163 excess visits (415.8 [95% CI, 396.4-426.2] visits per 1000 patients) for COVID-19; 6659 excess visits (52.1 [95% CI, 46.7-57.2] visits per 1000 patients) for dyspnea; 4565 excess visits (35.7 [95% CI, 29.5-41.7] visits per 1000 patients) for fever, malaise, or fatigue; 4289 excess visits (33.5 [95% CI, 29.3-37.7] visits per 1000 patients) for cough; and 3233 excess visits (25.3 [95% CI, 16.6-33.7] visits per 1000 patients) for arrythmias (Table 2). With the exception of cough, these PCCs and others were associated with notable excess burden in the inpatient or ED settings, in addition to outpatient and virtual care.

**Table 2.** Excess Health Care Utilization Associated With COVID-19 in 6 Months of Follow-up Among 255 718 Patients, March 15, 2020-May 15, 2021

Syndrome or classification	Overall		Inpatient		Emergency department		Outpatient		Virtual	
	Visits per 1000 patients (95% CI)^a^	Count^b^	Visits per 1000 patients (95% CI)^a^	Count^b^	Visits per 1000 patients (95% CI)^a^	Count^b^	Visits per 1000 patients (95% CI)^a^	Count^b^	Visits per 1000 patients (95% CI)^a^	Count^b^
All encounters	212.9 (146.5 to 278.4)	27 217	−0.9 (−5.6 to 3.5)	−116	14.7 (7.3 to 21.9)	1883	−62.8 (−111.2 to −15.1)	−8024	294.2 (255.8 to 332.0)	37 619
COVID-19	415.8 (396.4 to 426.2)	53 163	11.4 (9.3 to 12.0)	1456	19.4 (11.3 to 20.7)	2476	84.2 (68.4 to 91.3)	10760	304.3 (292.6 to 307.1)	38 907
Infectious disease sequelae	4.0 (3.2 to 4.0)	510	NA	0	NA	0	1.7 (0.5 to 1.8)	220	NA	0
Alopecia	14.5 (13.0 to 15.8)	1858	0.0 (−0.2 to 0.1)	0	NA	0	4.5 (3.5 to 5.2)	570	9.8 (8.2 to 11.1)	1259
Bronchitis	6.2 (5.2 to 7.1)	793	0.0 (−0.2 to 0.2)	5	0.5 (0.2 to 0.7)	61	0.8 (0.1 to 1.3)	98	4.9 (4.0 to 5.6)	625
Pulmonary edema/deep vein thrombosis	10.1 (6.3 to 13.0)	1289	0.9 (0.2 to 1.4)	121	1.1 (0.6 to 1.5)	142	4.9 (2.3 to 6.9)	631	3.5 (1.9 to 4.6)	452
Dyspnea	52.1 (46.7 to 57.2)	6659	1.3 (0.4 to 2.0)	161	6.5 (4.6 to 8.1)	828	19.9 (16.4 to 23.1)	2545	24.8 (21.7 to 27.5)	3170
Hypoxemia	14.4 (12.0 to 16.7)	1846	0.5 (0.0 to 0.9)	66	1.2 (0.6 to 1.7)	152	5.9 (4.2 to 7.4)	757	7.4 (5.6 to 8.9)	940
Cough	33.5 (29.3 to 37.7)	4289	−0.2 (−0.8 to 0.1)	−29	0.4 (−0.6 to 1.3)	56	11.5 (8.8 to 14)	1474	21.4 (18.3 to 24.3)	2739
Constitutional fever, malaise, or fatigue	35.7 (29.5 to 41.7)	4565	0.7 (−0.3 to 1.4)	85	1.4 (−0.2 to 2.8)	178	10.7 (6.4 to 14.5)	1367	25.2 (21.5 to 28.5)	3219
Pain in throat or chest	22.3 (17.0 to 27.2)	2853	to 0.4 (−1.5 to 0.4)	−57	6.3 (4.1 to 8.2)	800	8.1 (5.2 to 10.9)	1042	10.3 (7.7 to 12.6)	1320
Ear, nose, and throat disorders	15.6 (10.9 to 20.0)	1994	0.8 (0.0 to 1.4)	97	0.2 (−0.4 to 0.7)	31	8.6 (5.1 to 11.7)	1094	6.5 (3.5 to 9.1)	826
Arrhythmias	25.3 (16.6 to 33.7)	3233	0.5 (−1.3 to 2.1)	61	2.8 (1.1 to 4.4)	364	16.8 (10.7 to 22.3)	2146	6.9 (3.1 to 10.3)	884
Delirium or encephalopathy	4.7 (0.5 to 8.2)	597	−0.1 (−1.1 to 0.6)	−17	0.4 (−0.5 to 1.1)	55	2.9 (−0.1 to 5.3)	376	1.5 (−0.2 to 2.8)	191
Skin disorders	5.5 (2.0 to 8.9)	702	0.0 (−0.4 to 0.2)	−6	−0.2 (−1.0 to 0.6)	−21	2.9 (0.6 to 5.0)	372	3.4 (0.8 to 5.6)	429
Sleep disorders	9.2 (1.6 to 16.8)	1176	−0.3 (−1.8 to 0.9)	−42	0.2 (−0.6 to 0.9)	29	5.8 (0.3 to 10.8)	736	10.5 (5.9 to 14.7)	1347
Diabetes	23.1 (9.8 to 35.1)	2953	0.5 (−1.6 to 2.3)	61	0.7 (−1.5 to 2.6)	86	−3.9 (−13 to 4.9)	−499	14.1 (3.6 to 24.1)	1801
Headache	6.3 (0.1 to 11.9)	805	0.0 (−0.8 to 0.7)	1	−0.8 (−2.3 to 0.5)	−104	2.4 (−1.5 to 6.0)	302	7.5 (3.3 to 11.4)	963
Anxiety	17.1 (2.2 to 32.9)	2189	−1.0 − 2.3 to 0.2)	−123	0.8 (−0.7 to 2.1)	104	13.2 (6.7 to 19.1)	1684	3.4 (−13.7 to 19.5)	438
Myalgia or arthralgia	−21.7 (−35.6 to −9.8)	−2775	0.1 (−0.7 to 0.7)	8	−0.6 (−2.2 to 0.9)	−72	−22 (−33.3 to −11.3)	to 2819	9.1 (2.2 to 15.5)	1160
Nausea or vomiting	−4.9 (−8.7 to −1.2)	−625	−0.5 (−1.2 to 0.0)	−62	−1.7 (−3.3 to −0.4)	−223	−2.0 (−4.4 to 0.1)	−256	1.8 (0.0 to 3.4)	233
Stroke	−6.6 (−14.8 to −0.2)	−842	−0.8 (−2.3 to 0.4)	−98	0.0 (−0.9 to 0.6)	−5	−4.2 (−11.1 to 0.6)	−541	−0.6 (−2.9 to 0.9)	−78
Dementia	−9.1 (−17.8 to −1.9)	−1166	−1.7 (−3.6 to −0.3)	−221	−0.3 (−1.8 to 0.8)	−35	−5.4 (−12.3 to −0.2)	−690	−0.6 (−3.9 to 1.7)	−81
Lymphadenopathy	−2.5 (−5.1 to −0.4)	−323	0.0 (−0.4 to 0.2)	−2	0.0 (−0.4 to 0.2)	−4	−0.9 (−2.8 to 0.6)	−110	−1.8 (−3.9 to −0.5)	−230
Psychosis	−4.5 (−8.8 to −1.1)	−577	−0.2 (−1.0 to 0.3)	−21	−0.1 (−0.6 to 0.3)	−11	0.3 (−2.3 to 2.0)	37	−3.8 (−8.4 to −0.6)	−480

Abbreviation: NA, not applicable.

^a^ Excess utilization is not statistically significant if the 95% CI covers zero.

^b^ Visit counts across rows do not sum to the 27 217 total for all encounters as the 27 217 estimate for all encounters represents a net difference of encounters for all overall conditions, including those not in this table.

## Discussion

In this large, retrospective matched cohort study conducted across 8 large and diverse integrated health care systems, we found that having a SARS-CoV-2–positive test result was associated with an additional 213 health care visits per 1000 patients during the 6 months after the acute stage of illness. This volume of more than 27 200 visits among approximately 128 000 patients over 6 months demonstrates the potential for COVID-19 to exert an ongoing strain on health care organizations. This is particularly important in the context of deferred routine care since the inception of the pandemic.^22^

Several PCCs that were associated with elevated utilization were consistent with those identified by the CDC and others,^12,23,24^ including ENT disorders (such as loss of taste or smell), bronchitis, PE or DVT, chest pain, dyspnea, cough, and fatigue. These PCCs are consistent with previous long-term follow-up studies reporting the persistence of at least 1 COVID-19 symptom in more than 70% of patients, some for up to 6 months.^25^ Of all selected outcomes studied, COVID-19 and concerns or sequelae related to infectious disorders demonstrated the greatest increase in health care utilization associated with COVID-19 over 6 months; of the remaining 42 outcomes, the highest increase in utilization was observed for alopecia, bronchitis, PE or DVT, and dyspnea, which were each associated with approximately 2-fold the rate of utilization in patients with positive SARS-CoV-2 results compared with those with negative results over the same time period. These PCCs are consistent with other reports of patient-reported symptoms months after SARS-CoV-2 infection.^9,25,26^

We found moderate associations between COVID-19 and certain PCCs previously identified as some of the strongest associated PCCs, such as headache, sleep disorders, or anxiety.^9,12,24^ However, the lack of control groups in prior studies may have led to overestimation of these PCCs; estimates often reflect overall pandemic-related stressors felt by all, rather than those directly associated with SARS-CoV-2 infection. While some prior studies have attempted to control for these factors,^16,17^ the difference-in-difference approach used in this study robustly corrects for potential secular confounding.

The highest burden of care was observed during the initial 3 months postindex date, yet some PCCs persisted for 4 to 6 months after the index date, including alopecia, ENT disorders, arrythmias, and PE or DVT. In particular, alopecia demonstrated notable associations with COVID-19 up to 6 months after acute illness. Alopecia has been reported as associated with many communicable diseases, including acute^27,28^ and postacute COVID-19.^18,29^ This observation is thought to involve the role of androgens resulting from stress response.^2^ The elevated risk of PE or DVT also persisted for 6 months, at both early (0-3 months) and late (4-6 months) time periods. Although studies have identified PE or DVT as postacute sequelae of COVID-19, prior studies have lacked statistical power and long-term follow-up data.^18^ Notably, associations between PCCs related to the respiratory and circulatory system, such as bronchitis, dyspnea, cough, pain in throat or chest, and hypoxemia, that were associated with elevated utilization up to 3 months after acute COVID-19 infection were attenuated at 4 to 6 months. This observation has been reported previously (albeit among smaller populations without control populations)^12^ and is thought to represent lingering symptoms of acute illness.

In contrast, some PCCs worsened over time, including sleep, skin, and myoneural disorders, demonstrating that PCCs occurring at later time periods may include a broader range of conditions affecting multiple body systems compared with early time periods, as has been suggested elsewhere.^30^ Owing to the robust nature of the study design and data availability, this study represents an important data source to enhance our understanding of PCC. Prior PCC studies have been subject to a variety of shortfalls related to study design, often investigating specialty-specific outcomes only (eg, neurologic, cardiovascular) or achieving inadequate control for demographics, comorbidities, or changes in health care–seeking behaviors or exposure rates since the inception of the COVID-19 pandemic.^7,25,31^ Furthermore, large-scale studies have often relied on self-reported outcomes, which are subjective and prone to recall bias.^12,25^ Of the limited studies evaluating PCC from a health care utilization perspective, generalizability is limited owing to small sample sizes, no comparison group, or study populations limited to solely nonhospitalized^9^ or hospitalized patients.^8,32^ Our approach using a difference-in-difference design controls for individual-level characteristics as well as secular trends.

This study also represents one of the largest and most comprehensive studies of long COVID among children aged 0 to 17 years, with more than 25 000 children included. We found an overall reduction in health care utilization among children with SARS-CoV-2 infection during a 6-month follow-up period. However, children appeared to report a different pattern of PCCs compared with adults, with very few of the 44 PCCs studied demonstrating significant positive associations with COVID-19 after 6 months among children (COVID-19, PE or DVT, arrythmias, dyspnea, and ENT disorders). In particular, compared with negative controls, PE or DVT demonstrated more than 20-fold times higher utilization rates among children with positive SARS-CoV-2 test results, which could warrant further investigation, since these children appeared to have predisposing risk factors. Compared with adults, data on PCC among children are sparse. One of the earliest studies, a study by Buonsenso et al^33^ among 129 children in Italy, reported that 42.6% of children presented with at least 1 PCC up to 60 days after SARS-CoV-2 infection, with reported symptoms similar to those observed in adults. More recent studies have since suggested that most symptoms among children appear to resolve within 5 months.^34,35,36^ However, as the distribution of SARS-CoV-2 variants rapidly evolve, there is increasing concern about PCC in children, particularly given the low vaccination coverage among children worldwide.^36,37^ Further evidence is required to characterize PCC among children, since the syndrome list for this study was developed based on prior work among adults only. Indeed, for some PCC outcomes, only small numbers of events occurred among children, increasing the likelihood of spurious associations being identified. This may have explained the inverse association with dementia among children, which, after EMR review, was mostly associated head trauma and, hence, was unlikely to have been related to COVID-19.

### Limitations

There are limitations to this study. Our follow-up time is limited to 6 months, which may underestimate the burden of PCCs with later onsets or long symptomatic periods. We define our index date as 14 days after a SARS-CoV-2 test; while relevant from a health care utilization perspective, this definition may include visits associated with acute infection and hence may overestimate the health care burden due to postacute sequelae. Furthermore, we may underestimate excess utilization associated with COVID-19 as some of our populations with negative test results may have been tested for preprocedure clearance, which could be associated with higher health care utilization during follow up. It is also possible that patients were tested for COVID-19 outside of the health care organizations included here, which may have diluted the observed associations. We include persons with sustained membership to the included health care organizations; therefore, our findings may not represent the uninsured population. Furthermore, we may be underestimating the true burden of COVID-19 over 6 months owing to survival bias, since patients must have survived 6 months after acute infection to contribute data to the analysis. This may have explained the apparent protective association of COVID-19 for PCCs that are common in frail and multimorbid adult populations, such as dementia or stroke, in which we observed an inverse association after 6 months following acute illness. Importantly, a key assumption of the difference-in-difference approach is that trends in the outcome between groups would have been parallel in the absence of the exposure (COVID-19). However, consistent with prior work,^32^ the population with negative SARS-CoV-2 test results in this study had more comorbidities at baseline; therefore, they may have been more likely to seek care during the COVID-19 pandemic, which could attenuate observed effect estimates.^38^ Furthermore, we did not investigate time-varying treatment effects,^39^ as our study covers a short period of time; we do not expect the pathogenicity of COVID-19 to have changed substantially over the study period, particularly given that the study period ended prior to the predominance of the recent variants associated with enhanced disease severity. Additionally, owing to the extensive number of outcomes studied, spurious associations may be observed as a result of multiple testing.

## Conclusions

This large cohort study using data from 127 859 patients with SARS-CoV-2 infection with matched controls spanning 8 diverse US integrated health care organizations describes the burden of health care utilization occurring in the 6 months immediately following postacute SARS-CoV-2 infection, including in resource-intensive inpatient and ED settings. As health care systems evolve to maintain high-quality clinical care during a dynamic and ongoing global pandemic, these data provide valuable evidence to inform long-term strategic resource allocation for patients previously infected with SARS-CoV-2. We identified PCCs associated with early vs late periods following SARS-CoV-2 infection, as well as subgroups at highest risk, deepening clinical awareness of a highly complex condition. Together, our findings contribute reliable and informative estimates of PCC burden to broadly support health care planning, patient care and prognosis, public policy, as well as advancement of our scientific knowledge on the long-term burden of COVID-19.

## Notes

Supplement. **eTable 1.** All 44 Conditions and *International Statistical Classification of Diseases, Tenth Revision, Clinical Modification* (*ICD-10-CM*) Codes **eTable 2.** Health Care Utilization Associated With COVID-19 for Select Post-COVID Conditions (PCC): Patient With Positive SARS-CoV-2 Test Results vs Those With Negative Results **eFigure 1.** Health Care Utilization for Select Post–COVID-19 Conditions (PCC) by Time Interval After Index Date: Patient With Positive SARS-CoV-2 Test Results vs Those With Negative Results **eFigure 2.** Health Care Utilization for Select Post–COVID-19 Conditions (PCC) With and Without Censoring Follow-up at COVID-19 Vaccination **eFigure 3.** Health Care Utilization for Select Post–COVID-19 Conditions (PCC) Among Children Aged Younger Than 18 Years
