# Supplementary material for: Health Care Utilization in the 6 Months Following SARS-CoV-2 Infection
Source: JAMA Netw Open. 2022 Aug 12;5(8):e2225657. doi: 10.1001/jamanetworkopen.2022.25657 (PMC9375168; doi:10.1001/jamanetworkopen.2022.25657)
Supplement: Supplement. — eTable 1. All 44 Conditions and International Statistical Classification of Diseases, Tenth Revision, Clinical Modification (ICD-10-CM) Codes eTable 2. Health Care Utilization Associated With COVID-19 for Select Post-COVID Conditions (PCC): Patient With Positive SARS-CoV-2 Test Results vs Those With Negative Results eFigure 1. Health Care Utilization for Select Post–COVID-19 Conditions (PCC) by Time Interval After Index Date: Patient With Positive SARS-CoV-2 Test Results vs Those With Negative Results eFigure 2. Health Care Utilization for Select Post–COVID-19 Conditions (PCC) With and Without Censoring Follow-up at COVID-19 Vaccination eFigure 3. Health Care Utilization for Select Post–COVID-19 Conditions (PCC) Among Children Aged Younger Than 18 Years [file jamanetwopen-e2225657-s001.pdf]

Supplemental Online Content

Tartof SY, Malden DE, Liu ILA, et al. Health care utilization in the 6 months following SARS-CoV-2 infection. *JAMA Netw Open*. 2022;5(8):e2225657.
doi:10.1001/jamanetworkopen.2022.25657

eTable 1. All 44 Conditions and *International Statistical Classification of Diseases, Tenth Revision, Clinical Modification* (*ICD-10-CM*) Codes

eTable 2. Health Care Utilization Associated With COVID-19 for Select Post-COVID Conditions (PCC): Patient With Positive SARS-CoV-2 Test Results vs Those With Negative Results

eFigure 1. Health Care Utilization for Select Post–COVID-19 Conditions (PCC) by Time Interval After Index Date: Patient With Positive SARS-CoV-2 Test Results vs Those With Negative Results

eFigure 2. Health Care Utilization for Select Post–COVID-19 Conditions (PCC) With and Without Censoring Follow-up at COVID-19 Vaccination

eFigure 3. Health Care Utilization for Select Post–COVID-19 Conditions (PCC) Among Children Aged Younger Than 18 Years

This supplemental material has been provided by the authors to give readers additional information about their work.

© 2022 Tartof SY et al. *JAMA Network Open.*

eTable 1. All 44 Conditions and *International Statistical Classification of Diseases, Tenth Revision, Clinical Modification* (*ICD-10-CM*) Codes

Classification	ICD-10-CM code
Abdominal pain	R10
Anxiety	F40, F41, F42, F43, F44, F45, F48, R45
Arrhythmias	R00, I47, I48, I49
Ataxia/movement disorders	R26, R27, G26
Autonomic dysfunction	I95.1, I49, G90, R55
Bronchitis	J20, J40, J41, J42
Cardiac complications, non-arrhythmia (Myocarditis/Pericarditis)	I30, I40, I51.4, B33
Change in bowel habits	K58, K59, A08, A09, R19.4, R19.7
Cough	R05
COVID-19	U07.1, U09.9, J12.82, B97.29, B34.2, M35.81
Cytopenia	D69, D72.81
Delirium or encephalopathy	F05, R40.0, R41, R44
Dementia	F01, F02, F03, G31
Diabetes	E10, E11
Dyspnea	R06
Encephalitis	A85, A86, G04, G05, R29
Pulmonary embolism/Deep vein thrombosis (PE/DVT)	I26, I82
Mood disorders	F30, F31, F32, F33, F34, F38, F39
ENT-Disorders of ear , nose and throat disorders	H90, H91, H92, H93, J31, R43, R13
Ophthalmologic conditions following stroke	H53, H54
Constitutional fever/malaise/fatigue	R50, R61, R53, G93.3
Alopecia	L63, L65
Headache	G43, G44, R51
Hypoxemia	R09
Infectious disease sequelae	B94
Interstitial pulmonary disease (ILD)	J84
Lymphadenopathy	R59
Myalgia/arthralgia	M02, M25, M79
Myoneural Disorders	G72, M60
Nausea/vomiting	R11
Pain in throat or chest	R07
Skin disorders	B09, R20, R21, R23
Stroke	I64, I69, G45, G46, I60, I61, I62, I63
Parkinsonism and other extrapyramidal syndromes	G21, G24, G25
Peripheral nerve disorders	G50, G51, G52, G53, G54, G55, G56, G57, G58, G59, G61, G62, G64, G65
Pulmonary edema	J81
Psychosis	F20, F21, F22, F23, F24, F25, F28, F29
Sleep disorders	G47, F51
Stress cardiomyopathy	I51.81
Renal diseases	N18, N19
Seizures	G40, G41
Thyroid disorders	E03, E06
Vertigo	A88, H81, R42
Weight loss	R63, R64

© 2022 Tartof SY et al. *JAMA Network Open.*

eTable 2. Health Care Utilization Associated With COVID-19 for Select Post-COVID Conditions (PCC): Patient With Positive SARS-CoV-2 Test Results vs Those With Negative Results

	**Negative (N=127,859)**			**Positive (N=127,859)**			
Number of Visits	**2019**	**2020**	**Rate Ratio**	**2019**	**2020**	**Rate Ratio**	**Ratio of Rate Ratio (95% CI)**
COVID-19			87.2			1698.2	19.47 (10.47-36.22)
0	127837 (99.98%)	126071 (98.6%)		127828 (99.98%)	104568 (81.78%)		
1	14 (0.01%)	1170 (0.92%)		29 (0.02%)	12963 (10.14%)		
2	4 (0%)	322 (0.25%)		2 (0%)	4366 (3.41%)		
3+	4 (0%)	296 (0.23%)		0 (0%)	5962 (4.66%)		
Infectious disease sequelae			6.0			516.0	86.00 (5.07-1458.33)
0	127858 (100%)	127834 (99.98%)		127858 (100%)	127585 (99.79%)		
1	0 (0%)	15 (0.01%)		1 (0%)	174 (0.14%)		
2	1 (0%)	6 (0%)		0 (0%)	48 (0.04%)		
3+		4 (0%)			52 (0.04%)		
Alopecia			1.4			3.5	2.52 (2.17-2.92)
0	127202 (99.49%)	126977 (99.31%)		127224 (99.5%)	125614 (98.24%)		
1	500 (0.39%)	629 (0.49%)		476 (0.37%)	1674 (1.31%)		
2	96 (0.08%)	170 (0.13%)		108 (0.08%)	404 (0.32%)		
3+	61 (0.05%)	83 (0.06%)		51 (0.04%)	167 (0.13%)		
Bronchitis			0.3			0.6	1.85 (1.62-2.12)
0	125487 (98.14%)	127195 (99.48%)		125545 (98.19%)	126732 (99.12%)		
1	2006 (1.57%)	511 (0.4%)		1901 (1.49%)	818 (0.64%)		
2	284 (0.22%)	100 (0.08%)		327 (0.26%)	194 (0.15%)		
3+	82 (0.06%)	53 (0.04%)		86 (0.07%)	115 (0.09%)		
Pulmonary embolism/deep vein thrombosis			1.8			3.1	1.74 (1.36-2.23)
0	127451 (99.68%)	127201 (99.49%)		127531 (99.74%)	127102 (99.41%)		
1	172 (0.13%)	240 (0.19%)		128 (0.1%)	235 (0.18%)		
2	85 (0.07%)	129 (0.1%)		80 (0.06%)	143 (0.11%)		
3+	151 (0.12%)	289 (0.23%)		120 (0.09%)	379 (0.3%)		

© 2022 Tartof SY et al. *JAMA Network Open.*

Dyspnea			1.4			2.4	1.73 (1.61-1.86)
0	123232 (96.38%)	122426 (95.75%)		123897 (96.9%)	120547 (94.28%)		
1	3250 (2.54%)	3439 (2.69%)		2750 (2.15%)	4240 (3.32%)		
2	821 (0.64%)	1013 (0.79%)		691 (0.54%)	1461 (1.14%)		
3+	556 (0.43%)	981 (0.77%)		521 (0.41%)	1611 (1.26%)		
Hypoxemia			1.1			1.7	1.48 (1.37-1.60)
0	124418 (97.31%)	124434 (97.32%)		125150 (97.88%)	124262 (97.19%)		
1	2834 (2.22%)	2558 (2%)		2263 (1.77%)	2525 (1.97%)		
2	442 (0.35%)	517 (0.4%)		341 (0.27%)	643 (0.5%)		
3+	165 (0.13%)	350 (0.27%)		105 (0.08%)	429 (0.34%)		
Cough			0.8			1.2	1.45 (1.37-1.53)
0	119081 (93.13%)	121437 (94.98%)		119738 (93.65%)	119933 (93.8%)		
1	6458 (5.05%)	4303 (3.37%)		6032 (4.72%)	5033 (3.94%)		
2	1563 (1.22%)	1289 (1.01%)		1412 (1.1%)	1659 (1.3%)		
3+	757 (0.59%)	830 (0.65%)		677 (0.53%)	1234 (0.97%)		
Constitutional-Fever/malaise/fatigue			1.4			2.0	1.41 (1.31-1.51)
0	121633 (95.13%)	120796 (94.48%)		122549 (95.85%)	119815 (93.71%)		
1	4626 (3.62%)	4688 (3.67%)		4048 (3.17%)	5201 (4.07%)		
2	977 (0.76%)	1277 (1%)		763 (0.6%)	1459 (1.14%)		
3+	623 (0.49%)	1098 (0.86%)		499 (0.39%)	1384 (1.08%)		
Pain in throat or chest			1.2			1.5	1.28 (1.20-1.36)
0	121656 (95.15%)	121265 (94.84%)		122479 (95.79%)	120677 (94.38%)		
1	4085 (3.19%)	4163 (3.26%)		3636 (2.84%)	4388 (3.43%)		
2	1265 (0.99%)	1330 (1.04%)		1039 (0.81%)	1560 (1.22%)		
3+	853 (0.67%)	1101 (0.86%)		705 (0.55%)	1234 (0.97%)		
Ear, nose and throat disorders			1.2			1.5	1.23(1.15-1.32)
0	122417 (95.74%)	121690 (95.18%)		123506 (96.6%)	122084 (95.48%)		
1	3606 (2.82%)	3882 (3.04%)		3034 (2.37%)	3653 (2.86%)		
2	1095 (0.86%)	1301 (1.02%)		808 (0.63%)	1205 (0.94%)		
3+	741 (0.58%)	986 (0.77%)		511 (0.4%)	917 (0.72%)		
Delirium or encephalopathy			1.6			1.9	1.22 (1.02-1.47)
0	126673 (99.07%)	126319 (98.8%)		126916 (99.26%)	126385 (98.85%)		

© 2022 Tartof SY et al. *JAMA Network Open.*

1	793 (0.62%)	914 (0.71%)		614 (0.48%)	860 (0.67%)		
2	217 (0.17%)	317 (0.25%)		184 (0.14%)	312 (0.24%)		
3+	176 (0.14%)	309 (0.24%)		145 (0.11%)	302 (0.24%)		
Arrhythmias			1.4			1.7	1.22 (1.14-1.32)
0	123089 (96.27%)	122156 (95.54%)		124037 (97.01%)	122426 (95.75%)		
1	2424 (1.9%)	2612 (2.04%)		1964 (1.54%)	2438 (1.91%)		
2	982 (0.77%)	1174 (0.92%)		774 (0.61%)	1152 (0.9%)		
3+	1364 (1.07%)	1917 (1.5%)		1084 (0.85%)	1843 (1.44%)		
Skin disorders			1.1			1.3	1.12 (1.04-1.21)
0	123265 (96.41%)	123286 (96.42%)		123954 (96.95%)	123505 (96.59%)		
1	3630 (2.84%)	3369 (2.63%)		3073 (2.4%)	3229 (2.53%)		
2	653 (0.51%)	734 (0.57%)		577 (0.45%)	710 (0.56%)		
3+	311 (0.24%)	470 (0.37%)		255 (0.2%)	415 (0.32%)		
Sleep disorders			1.3			1.4	1.07 (1.01-1.13)
0	119809 (93.7%)	118982 (93.06%)		121595 (95.1%)	120263 (94.06%)		
1	4645 (3.63%)	4730 (3.7%)		3594 (2.81%)	4170 (3.26%)		
2	1589 (1.24%)	1755 (1.37%)		1252 (0.98%)	1447 (1.13%)		
3+	1816 (1.42%)	2392 (1.87%)		1418 (1.11%)	1979 (1.55%)		
Diabetes			1.2			1.3	1.06 (1.02-1.09)
0	116179 (90.86%)	115991 (90.72%)		114997 (89.94%)	114075 (89.22%)		
1	3573 (2.79%)	3304 (2.58%)		4305 (3.37%)	4120 (3.22%)		
2	2442 (1.91%)	2323 (1.82%)		2831 (2.21%)	2807 (2.2%)		
3+	5665 (4.43%)	6241 (4.88%)		5726 (4.48%)	6857 (5.36%)		
Headache			1.1			1.2	1.06 (1.00-1.11)
0	119328 (93.33%)	118548 (92.72%)		120352 (94.13%)	119531 (93.49%)		
1	5406 (4.23%)	5748 (4.5%)		4959 (3.88%)	5183 (4.05%)		
2	1767 (1.38%)	1928 (1.51%)		1458 (1.14%)	1696 (1.33%)		
3+	1358 (1.06%)	1635 (1.28%)		1090 (0.85%)	1449 (1.13%)		
Anxiety			1.4			1.4	1.05 (1.01-1.10)
0	112310 (87.84%)	110352 (86.31%)		116944 (91.46%)	114581 (89.62%)		
1	7071 (5.53%)	7080 (5.54%)		5404 (4.23%)	5895 (4.61%)		
2	2890 (2.26%)	3129 (2.45%)		2099 (1.64%)	2426 (1.9%)		
3+	5588 (4.37%)	7298 (5.71%)		3412 (2.67%)	4957 (3.88%)		

© 2022 Tartof SY et al. *JAMA Network Open.*

Myalgia/arthralgia			1.2			1.1	0.94 (0.90-0.97)
0	108666 (84.99%)	108807 (85.1%)		111362 (87.1%)	111641 (87.32%)		
1	10246 (8.01%)	9405 (7.36%)		9356 (7.32%)	8482 (6.63%)		
2	3961 (3.1%)	3904 (3.05%)		3354 (2.62%)	3361 (2.63%)		
3+	4986 (3.9%)	5743 (4.49%)		3787 (2.96%)	4375 (3.42%)		
Nausea/vomiting			1.1			1.0	0.89 (0.82-0.97)
0	123513 (96.6%)	123759 (96.79%)		124247 (97.18%)	124709 (97.54%)		
1	3345 (2.62%)	2846 (2.23%)		2867 (2.24%)	2305 (1.8%)		
2	619 (0.48%)	702 (0.55%)		502 (0.39%)	510 (0.4%)		
3+	382 (0.3%)	552 (0.43%)		243 (0.19%)	335 (0.26%)		
Stroke			1.8			1.4	0.78 (0.61-0.99)
0	127158 (99.45%)	126909 (99.26%)		127212 (99.49%)	127103 (99.41%)		
1	301 (0.24%)	356 (0.28%)		278 (0.22%)	295 (0.23%)		
2	143 (0.11%)	169 (0.13%)		144 (0.11%)	146 (0.11%)		
3+	257 (0.2%)	425 (0.33%)		225 (0.18%)	315 (0.25%)		
Dementia			2.1			1.6	0.77 (0.63-0.94)
0	127275 (99.54%)	126929 (99.27%)		127102 (99.41%)	126742 (99.13%)		
1	285 (0.22%)	383 (0.3%)		315 (0.25%)	443 (0.35%)		
2	110 (0.09%)	199 (0.16%)		163 (0.13%)	245 (0.19%)		
3+	189 (0.15%)	348 (0.27%)		279 (0.22%)	429 (0.34%)		
Lymphadenopathy			1.5			1.1	0.76 (0.61-0.95)
0	127228 (99.51%)	127087 (99.4%)		127337 (99.59%)	127332 (99.59%)		
1	464 (0.36%)	495 (0.39%)		371 (0.29%)	368 (0.29%)		
2	109 (0.09%)	128 (0.1%)		96 (0.08%)	82 (0.06%)		
3+	58 (0.05%)	149 (0.12%)		55 (0.04%)	77 (0.06%)		
Psychosis			1.7			1.2	0.74 (0.59-0.92)
0	127406 (99.65%)	127217 (99.5%)		127539 (99.75%)	127494 (99.71%)		
1	154 (0.12%)	202 (0.16%)		122 (0.1%)	151 (0.12%)		
2	92 (0.07%)	103 (0.08%)		75 (0.06%)	51 (0.04%)		
3+	207 (0.16%)	337 (0.26%)		123 (0.1%)	163 (0.13%)		

PCC = Post-COVID Conditions, RRR= Relative Rate Ratio, CI= Confidence Interval.

*PCC outcomes presented are limited to those conditions that were statistically significant (24 of 44 conditions that were evaluated).

© 2022 Tartof SY et al. *JAMA Network Open.*

eFigure 1. Health Care Utilization for Select Post–COVID-19 Conditions (PCC)** by Time Interval After Index Date: Patient With Positive SARS-CoV-2 Test Results vs Those With Negative Results

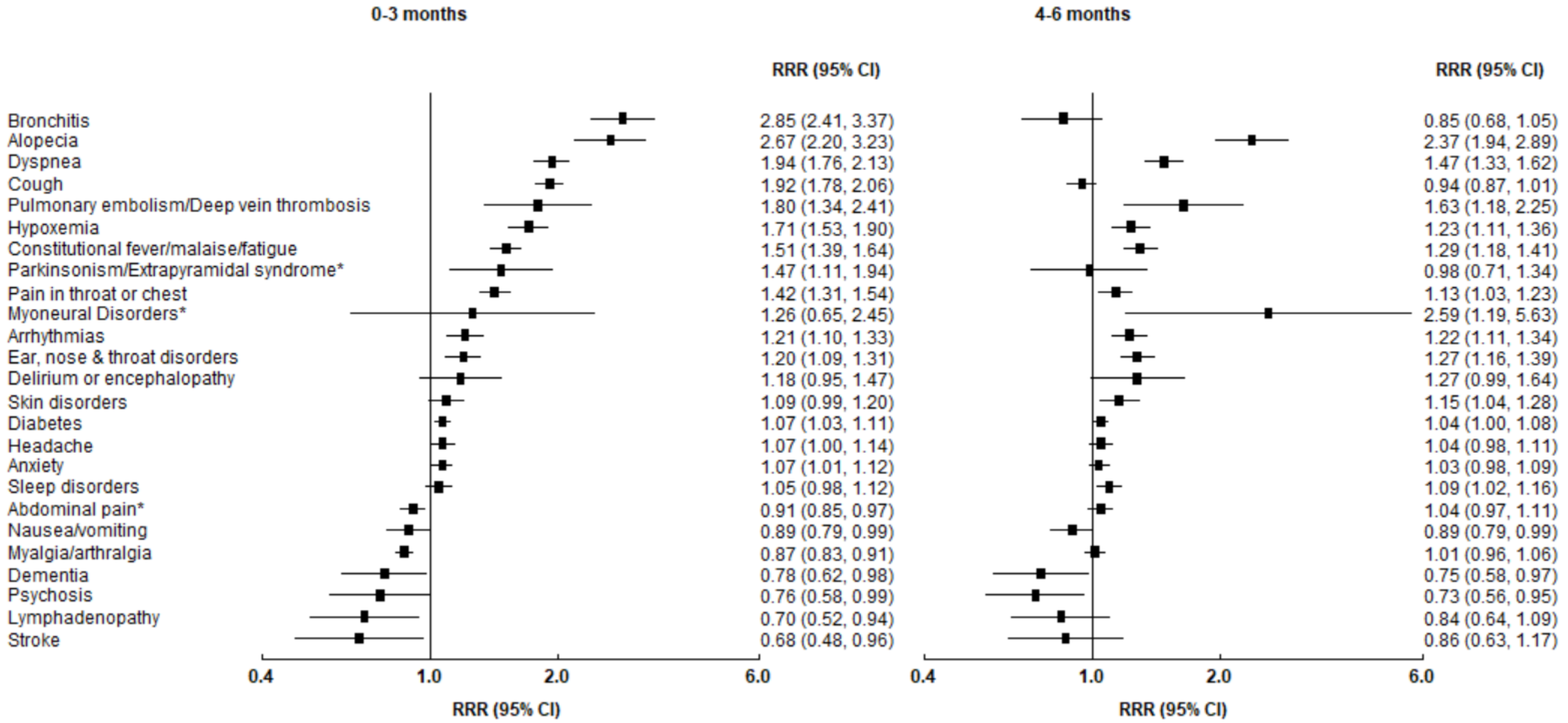

PCC = Post-COVID Conditions, RRR= Relative Rate Ratio, CI= Confidence Interval.
*PCC category was not significant in combined analyses, but was significantly associated with COVID-19 diagnosis in time-stratified analyses (0-3 months or 4-6 months).
**PCC categories presented are limited to those conditions that were statistically significant either in the overall findings or in time-stratified analyses (0-3 months or 4-6 months), excluding COVID-19 and infectious disease sequelae due to scale.

© 2022 Tartof SY et al. *JAMA Network Open.*

eFigure 2. Health Care Utilization for Select Post–COVID-19 Conditions (PCC)* With and Without Censoring Follow-up at COVID-19 Vaccination

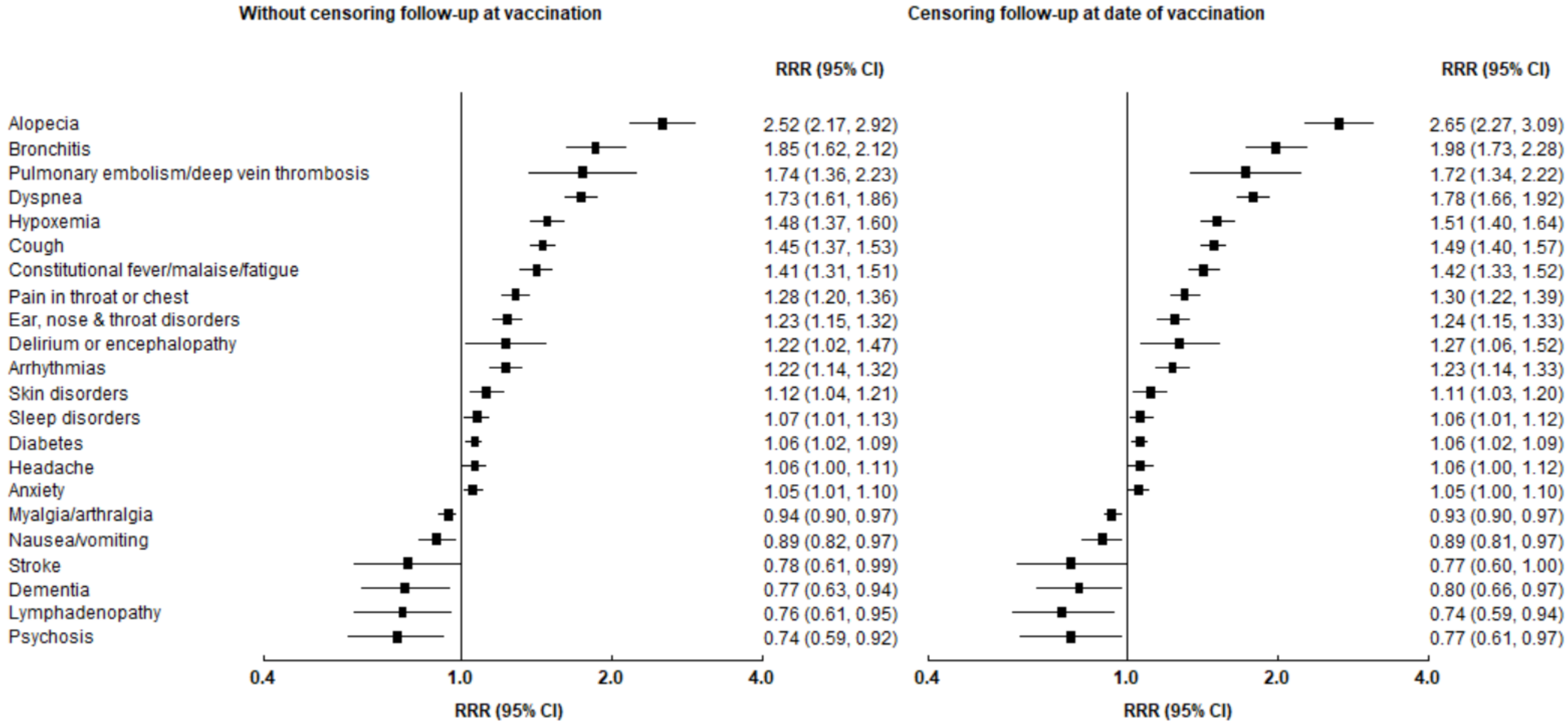

PCC = Post-COVID Conditions, RRR= Relative Rate Ratio, CI= Confidence Interval.
*PCC outcomes presented are limited to those conditions that were statistically significant in the overall findings, excluding COVID-19 and infectious disease sequelae due to scale.

© 2022 Tartof SY et al. *JAMA Network Open.*

eFigure 3. Health Care Utilization for Select Post–COVID-19 Conditions (PCC)* Among Children Aged Younger Than 18 Years

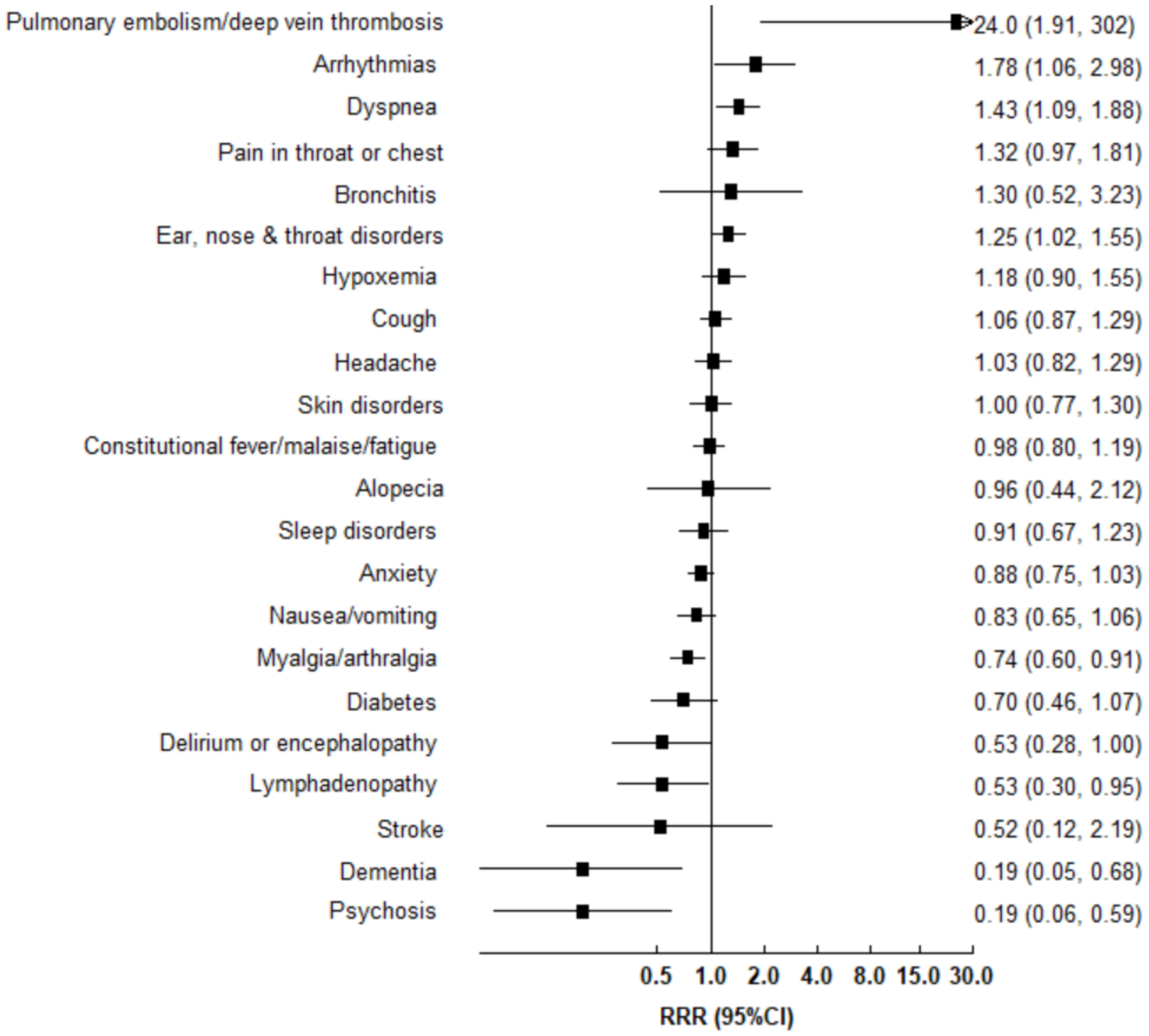

PCC = Post-COVID Conditions, RRR= Relative Rate Ratio, CI= Confidence Interval.
*PCC outcomes presented are limited to those conditions that were either statistically significant overall or among children < 18 years. There was an insufficient number of events to calculate the RRR associated with infectious disease sequelae among children aged <18 years.

© 2022 Tartof SY et al. *JAMA Network Open.*
